# The Recovery of Plastid Function Is Required for Optimal Response to Low Temperatures in *Arabidopsis*

**DOI:** 10.1371/journal.pone.0138010

**Published:** 2015-09-14

**Authors:** Peter Kindgren, Carole Dubreuil, Åsa Strand

**Affiliations:** Umeå Plant Science Centre, Department of Plant Physiology, Umeå University, Umeå, Sweden; Iwate University, JAPAN

## Abstract

Cold acclimation is an essential response in higher plants to survive freezing temperatures. Here, we report that two independent mutant alleles of the H-subunit of Mg-chelatase, CHLH, *gun5-1* and *cch* in *Arabidopsis* are sensitive to low temperatures. Plants were grown in photoperiodic conditions and exposed to low temperatures for short- and long-term periods. Tetrapyrrole biosynthesis was initially significantly inhibited in response to low temperature but recovered in wild type (Col-0), although the tetrapyrrole levels were lower in cold compared to control conditions. The *gun5-1* and *cch* alleles showed an inability to recover chlorophyll biosynthesis in addition to a significant decrease in freezing tolerance. We found that the impaired plastid function in the CHLH mutant plants resulted in compromised *de novo* protein synthesis at low temperatures. The expression of the transcription factors *CBF1-3* was super-induced in *gun5-1* and *cch* mutant alleles but expression levels of their target genes, *COR15a*, *COR47* and *COR78* were similar or even lower compared to Col-0. In addition, the protein levels of COR15a were lower in *gun5-1* and *cch* and a general defect in protein synthesis could be seen in the *gun5-1* mutant following a 35S labelling experiment performed at low temperature. Taken together, our results demonstrate the importance of a functional chloroplast for the cold acclimation process and further suggest that impaired plastid function could result in inhibition of protein synthesis at low temperature.

## Introduction

Changes in ambient temperatures affect growth and survival of most living organisms. To overcome any negative effect of exposure to low temperatures, many species have evolved an adaptive response named cold acclimation [1,2]. Cold acclimation enables an organism to survive freezing temperatures if they first are exposed to low non-freezing temperatures for a period of time. In higher plants, cold acclimation is very complex and comprises a multitude of changes to the metabolism of the plant [2]. A massive change in gene transcription is an important component of the cold acclimation process and in the model plant *Arabidopsis* around 1000 genes have been found to be differentially expressed following cold exposure [2]. A sub-set of these genes, termed cold responsive (*COR*), has been found to be highly up-regulated in response to low temperatures. The COR proteins have essential roles for plant survival in chilling and freezing temperatures [3]. Many COR genes are regulated via a cis-element (CCGAC, C-repeat or dehydration responsive element) in their promoter that is recognised by the C/DRE binding factors (CBF/DREB, hereafter called CBF). The *CBF1-3* genes are rapid but transiently induced following exposure to low temperatures and CBF1-3 are key transcription factors in the nucleus, not only involved in cold acclimation, but also in other plant stress responses [4]. In addition to stress regulation, the expression of *CBF1-3* is controlled by circadian- [5], light- [6] and plastid signals [7]. It has been suggested that chloroplasts could act as sensors of changes in temperature [8,9]. It is, however, unclear how the chloroplasts relay this information to the rest of the cell to trigger the acclimation response.

Plastid or retrograde signals originate in the chloroplast and regulate nuclear gene expression and a number of different plastid signals with specific regulons have been identified (for review, see [10]). The first mutants isolated impaired in plastid-to-nucleus signalling were the genome uncoupled (*gun*) mutants [11]. The *gun* mutants display impaired repression of nuclear encoded photosynthesis genes in response to inhibition of plastid development; hence expression of the nuclear genes is uncoupled from the status of the plastid. Out of six isolated mutants, five have mutations in various enzymes in the plastid localized tetrapyrrole pathway [12–14]. One of them, *gun5*, has a mutation in the H subunit of the first unique enzyme of the chlorophyll branch, magnesium chelatase (CHLH). The product of Mg-chelatase, magnesium protoporphyrin IX (Mg-ProtoIX) was identified as a potential signalling molecule connecting the activities of the chloroplast with the nucleus [15]. The signalling role of Mg-ProtoIX was questioned when its steady state levels were found to not correlate with nuclear photosynthetic gene expression [16,17]. Whether accumulation of Mg-ProtoIX is itself an important part of the tetrapyrrole-mediated signal remains unclear but it is evident that impaired flux through chlorophyll biosynthesis and the accumulation of Mg-ProtoIX/Mg-ProtoIX-ME is an indicator of changes in the environment and results in changes in nuclear gene expression. In addition, there is an established genetic link between CHLH and cytosolic and nuclear factors controlling nuclear gene expression [18,19] although the exact mode-of-action of CHLH in plastid-to-nucleus signalling remains elusive.

The Mg-chelatase complex comprises of three nuclear encoded proteins; the ligand binding H-, the ATPase I-, and metal ion coordinator D-subunit [20,21]. In addition, CHLH is regulated by GUN4, a porphyrin binding protein that assists the association between CHLH and the membrane [22]. Intriguingly, CHLH is one of the proteins in higher plants with the shortest half-life, comparable to that of the core subunits of PSII [23], which suggests that it is one of the primary regulatory steps in the tetrapyrrole pathway. Distinct from its role in chlorophyll biosynthesis and retrograde signalling, CHLH is a putative abscisic acid (ABA) receptor in *Arabidopsis* [24]. The possible role as an ABA receptor suggests that CHLH is able to traverse the envelope membrane and published reports show that both the N- and C-terminal part of CHLH have a cytosolic localisation when inserted in the chloroplast envelope membrane [19]. The association of CHLH to the chloroplast envelope membrane presents CHLH in a unique position to possibly sense changes in membrane fluidity following changes in temperature. Here, we report that a fully functional CHLH is required for optimal cold acclimation in *Arabidopsis*. We show that two mutant alleles of CHLH, *gun5-1* and *c*onditional *ch*lorina (*cch*) [14], have an impaired ability to acclimate to low temperatures and survive freezing temperatures. The cold acclimation is accompanied with reduced protein translation during low temperatures in the CHLH mutants. Our results demonstrate the importance of a functional chloroplast and the recovery of photosynthetic activity for the cold acclimation process.

## Material and Methods

### Plant material, growth conditions and TEM microscopy

*Arabidopsis* plants were grown for 5 weeks on soil in short day conditions (9 hours light/15 hours dark period, 22°C/18°C, 150 μmol photons m^-2^ s^-1^, control conditions). The *gun5-1* mutant and the *cch* mutant used are in the Colombia background (Col-0) and described elsewhere [14]. For cold treatment, plants were transferred to 4°C and identical light regime, or 25 μmol photons m^-2^ s^-1^. Samples were collected at the indicated times as seen in the figures. For TEM microscopy, samples were taken from 5 week old plants grown in control conditions and after 28 days of cold treatment. The preparation, embedding and cutting were done according to Keskitalo et al. [25].

### Western blotting

Protein extracts from 5 week old plants were separated on a SDS-PAGE gel and blotted according to [26]. The GUN4 antibody [27] and the COR15a antibody [28] were kindly given by the authors. All other antibodies used were ordered and used in dilutions as per instructions from the manufacturer (Agrisera).

### RNA extraction, cDNA synthesis and qPCR

RNA was extracted from leaf tissue of 5 week old plants (control) or cold treated plants. Total RNA was used for DNase treatment (Ambion Turbo-DNAse) and subsequently used for cDNA synthesis (Bio-Rad, iScript cDNA Synthesis Kit). cDNA in a 1 to 20 dilution was mixed with primers (final concentration 0.5 μM) and iQ SYBR Green Supermix (Bio-Rad). Reactions were run in triplicates in a CFX96 real-time system (Bio-Rad) and monitored with CFX manager (Bio-Rad). Data was analysed as described [29]. Primers used in this study can be found in S1 Table.

### Sugar, chlorophyll and tetrapyrrole measurements

Soluble sugars were measured with an enzyme-coupled assay described elsewhere [30,31].

Samples for chlorophyll measurements were frozen in liquid nitrogen and homogenised in 80% buffered acetone (80% Acetone, 25 mM Hepes pH 7.5). Samples were measured in 1 cm glass cuvette with a spectrophotometer by 646.6 nm, 663.6 nm and 750 nm, using buffered acetone as blank [32].

For tetrapyrrole levels, leaf samples were collected and homogenised in acetone: 0.1 M NH_4_OH (8:2 v/v) and identified and quantified using authentic standards from Frontier Scientific. The tetrapyrrole levels were monitored with HPLC according to the method used by Mochizuki et al (2008).

### Freezing test

Leaf discs of 5 week old non-acclimated or cold-acclimated (3 days in 4°C) plants were put in glass tubes (two in each tube, each one cm^2^) with 200 μl of deionized water (HPLC-grade). Both leaf discs were in contact with the water. The tubes were then transferred to a programmable ethanol bath at -2.5°C (Julabo FP45, Germany). After 1 hour, ice formation was induced by a metal stick frozen in liquid nitrogen and the temperature was slowly decreased (-2°C/h). Samples were taken out of the bath at designated temperatures and cooled on ice for an hour followed by 4°C. When all samples were collected, 1.3 ml of deionized water was added and the tubes were shaken overnight at 4°C. Electrolyte leakage was measured using a conductivity cell (CDM210, Radiometer, Copenhagen, Denmark). To get total ion content, tubes were immersed in liquid nitrogen, thawed, shaken again overnight and measured for conductivity. Electrolyte leakage was determined by comparing the measured conductivity before and after the liquid nitrogen treatment. Data was fitted to a sigmoidal dose-response with GraphPad Prism.

### Labelling with ^35^S Amino Acids

Labelling experiments were done according to Guo et al (2002) with a few modifications. 3 week old plants grown on 1×MS, 1% sucrose plates were pre-treated at 0°C for 4 days and seedlings were then painted with labelling mix [33]. Seedlings were then transferred to either 22°C or back to 0°C for 36 hours. After harvest, seedlings were briefly rinsed with water and proteins extracted and run on a denaturing gel. The gel was then stained, dried and exposed to an X-ray film. The X-ray film was subsequently scanned and the image analysed in ImageJ. The overall area of the 35S signal was calculated as the total area subtracted to the adjacent background and normalized to the area of the Coomassie stain. The relative 35S signal strength between Col-0 and the *gun5* mutant was calculated from 3 independently prepared samples as the ratio (Col-0/*gun5*) of the normalized areas.

## Results

### The *gun5* mutant has impaired ability to acclimate to low temperatures

**Fig 1:**
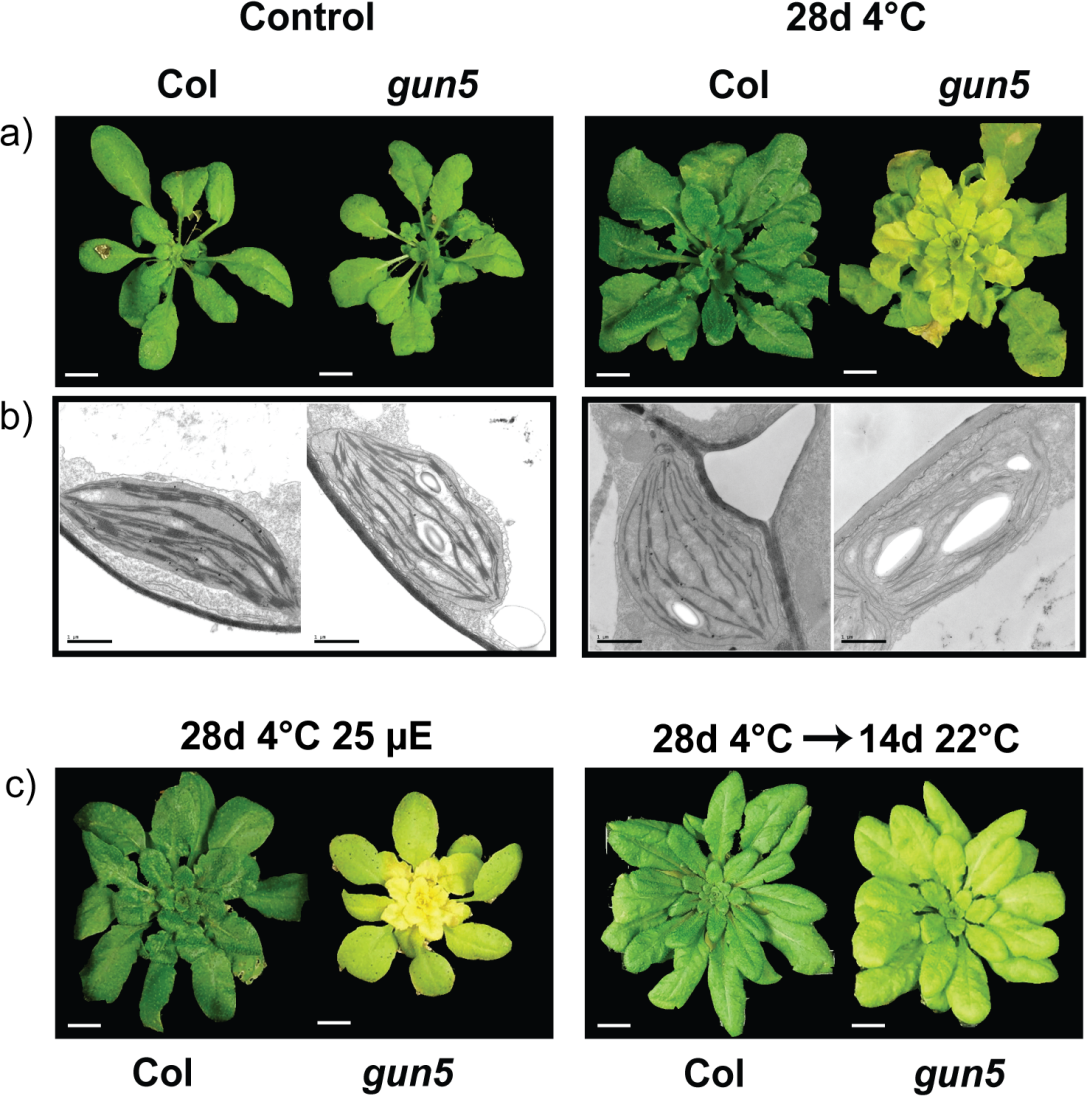
The *gun5* mutant shows an enhanced but reversible phenotype after long term exposure to low temperatures. (a) 5 week old plants grown in short day condition (9/15 h light/dark, 22°C/18°C, 150 μE, control conditions) were transferred to 4°C, short day condition (9/15 h light/dark, 4°C/4°C, 150 μE) for 28 days. Bar represents 1 cm. (b) Transmission electron microscopy images of plastids from corresponding plants above. Bar represents 1 μm. (c) 5 week old plants were grown in SD conditions (150 μE) and transferred to SD conditions, 4°C (9/15 h light/dark, 4°C/4°C, 25 μE). After 28 days of cold exposure, plants were transferred back to control conditions for an additional 14 days for recovery.

**Fig 2:**
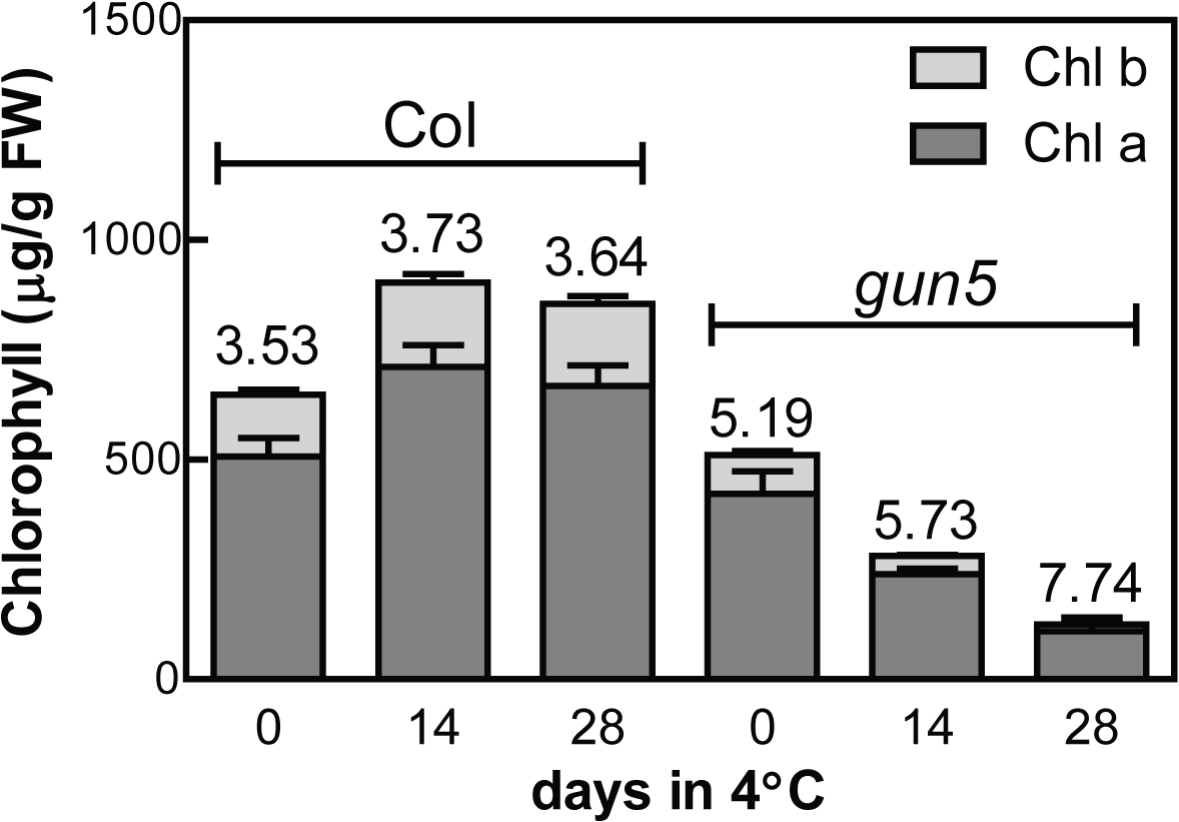
Chlorophyll content in Col-0 and *gun5* plants following exposure to low temperatures. Samples were taken from 5 week old plants grown under short day conditions (time point 0) and after indicated days grown in 4°C, short days conditions. Above each column is the Chl a/b ratio from each sample. Values represent at least 6 biological replicates (±SD).

The *gun5-1* mutant (hereafter called *gun5*) has a point mutation in the H-subunit of Mg-chelatase, CHLH, the first enzyme in the pathway that commits tetrapyrroles to the biosynthesis of chlorophyll. The pale leaves of *gun5* are caused by a reduced flux through the tetrapyrrole pathway resulting in lower amount of chlorophyll [14]. Interestingly, when the *gun5* mutant was exposed to low temperatures, the pale phenotype was significantly enhanced (Fig 1A). All new leaves formed in the *gun5* mutant under low temperatures showed a striking pale appearance. The opposite could be seen in wild type where the new leaves showed a darker green phenotype compared to the warm grown control. Electron microscope images of the chloroplasts confirmed that chloroplast structure was affected in *gun5* following exposure to low temperature (Fig 1B). Wild type chloroplasts showed somewhat reduced grana stacks following exposure to low temperature compared to control conditions, but the *gun5* mutant displayed severely disrupted thylakoid membrane structures following cold exposure. The pale phenotype in response to low temperatures could also be seen when grown under very low light, ruling out a possible effect of oxidative stress on chlorophyll biosynthesis and/or accumulation (Fig 1C). In addition, when the *gun5* plants were transferred back to the control conditions the low temperature induced pale phenotype was recovered (Fig 1C). The chlorophyll levels in response to low temperature confirm the visible phenotype for both genotypes, wild type showed an increase in total chlorophyll and the chlorophyll a/b ratio while the *gun5* mutant displayed a sharp decrease in total chlorophyll content and an increased chlorophyll a/b ratio (Fig 2).

### Photosynthetic gene expression and protein levels in the *gun5* mutant in response to low temperatures

**Fig 3:**
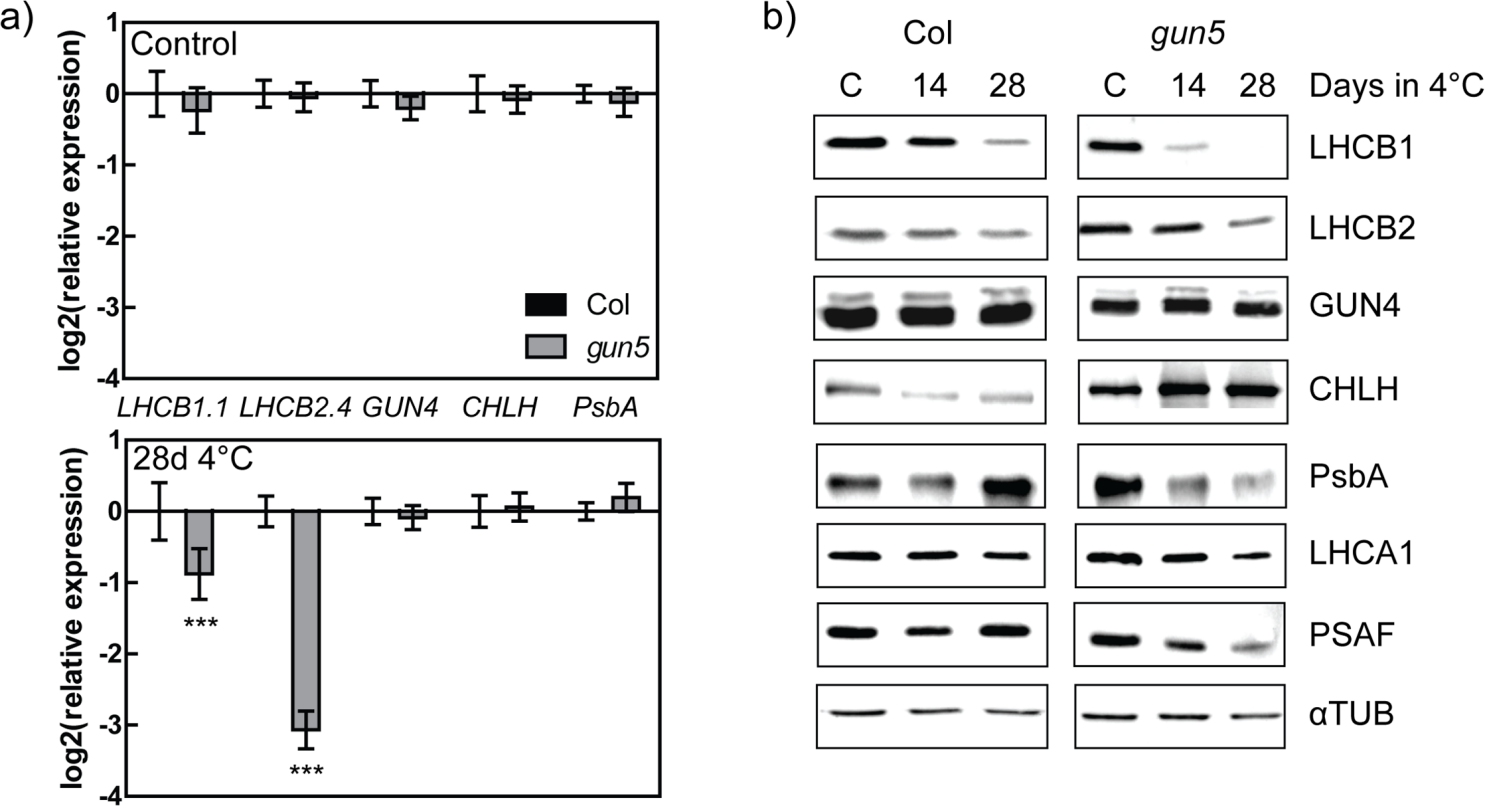
Gene expression and protein levels in Col-0 and the *gun5* mutant following exposure to low temperatures. (a) Expression of photosynthetic associated genes in *gun5* relative to Col-0 in 5 week old plants grown in control conditions and after 28 days in low temperature (4°C). The expression was normalized to Ubiquitin like protein (At4g36800) and related to the amount present in Col-0. Each data point represents values from 3 biological replicates (±SD). Significant differences were found with Two-way ANOVA and Bonferroni post-test, p<0.001 (***). (b) Protein levels of photosynthetic proteins in Col-0 and *gun5* in control condition and following 14 and 28 days exposure to low temperature. αTUB was used as a loading control.

In order to understand the reason for the enhanced pale phenotype of the *gun5* mutant when exposed to low temperatures, we investigated the expression and protein levels of genes and proteins involved in photosynthesis in control plants and plants exposed to low temperatures for 14 and 28 days. Expression of both nuclear and plastid encoded genes was similar to wild type in the *gun5* mutant when the plants were grown under warm control conditions (Fig 3A), as were the protein levels (Fig 3B). Following extended exposure to low temperature, the *gun5* mutant showed lower expression levels of *LHCB1*.*1* and *LHCB2*.*4* compared to wild type while *GUN4*, *CHLH* and the plastid encoded *PsbA* expression was similar to what was found in wild type. The difference in expression levels was also partly reflected in the amount of the respective proteins. Following exposure to low temperature wild type decreased the levels of light harvesting proteins, especially LHCB1, and increased the levels of core photosystem proteins (Fig 3B). The *gun5* mutant showed an overall decrease in the levels of photosynthetic proteins compared to wild type with the exception of GUN4 and CHLH which showed a steady or increased level, respectively, presumably in an attempt to recover the impaired chlorophyll biosynthesis in *gun5*.

### Tetrapyrrole levels abruptly decline in response to low temperatures

**Fig 4:**
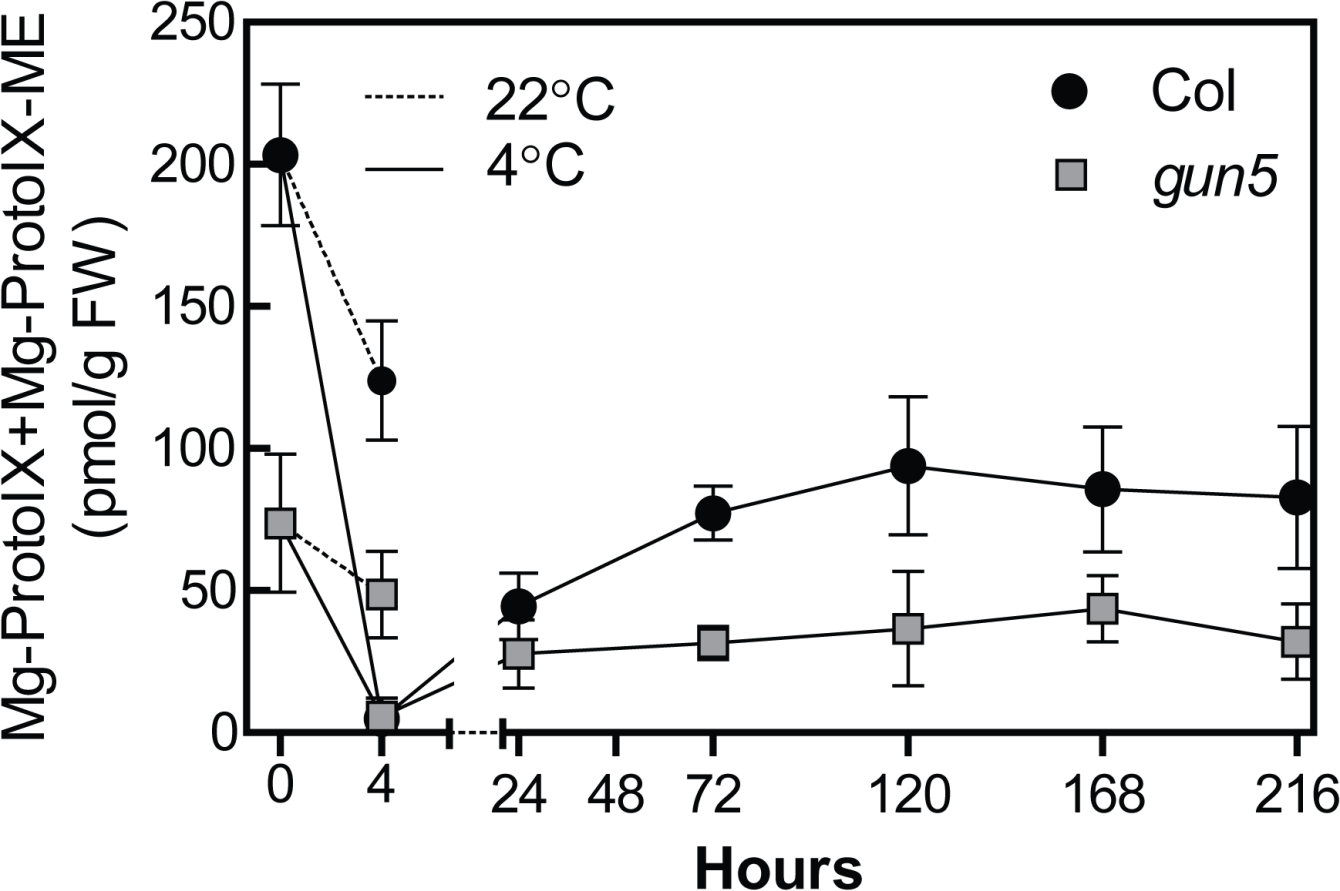
Levels of Mg-ProtoIX and Mg-ProtoIX-ME in Col-0 and *gun5* following exposure to low temperatures. Plants were grown for 5 weeks in SD conditions and transferred to cold conditions and sampled after indicated time points where time point 0 is the middle of the day. The first sample (4h) was taken at the end of the light period of the first day. Each data point represents at least 3 biological replicates (±SD).

To investigate the flux through the tetrapyrrole pathway in response to exposure to low temperatures we determined the levels of the chlorophyll intermediates Mg-ProtoIX and Mg-ProtoIX-ME in the *gun5* mutant and wild type following short- and medium term exposure to low temperature. Five week old plants grown in short day conditions were transferred to 4°C at the middle of the day. The plants were still kept under a short day light regime and samples were collected at time points indicated in Fig 4. Already at the end of the first day (4 hours after the transfer to 4°C), Mg-ProtoIX and Mg-ProtoIX-ME levels decreased to almost undetectable levels in both wild type and the *gun5* mutant (Fig 4), indicating a very rapid and strong inhibition of the tetrapyrrole pathway following a shift to low temperatures. The rapid decline of tetrapyrroles was not seen in control conditions (Fig 4). This result is consistent with earlier reports demonstrating that the enzymes in the chlorophyll biosynthetic pathway are strongly inhibited by low temperatures resulting in an inactivation of the pathway [34]. In wild type, the tetrapyrrole levels slowly recovered from the initial decrease to reach a steady level after 5 days, representing 40–50% of the levels detected in the control samples (Fig 4). In the *gun5* mutant, tetrapyrrole levels also recovered but reached considerably lower level compared to wild type after 5 days in the cold. This is also seen in the control plants where *gun5* only contained about 30–40% of the wild type levels of the tetrapyrroles. Possibly the additional inhibition on Mg-chelatase activity in the *gun5* mutant caused by low temperature generates an inhibition so strong that chlorophyll biosynthesis simply cannot proceed.

### Freezing tolerance is affected in CHLH mutants

**Fig 5:**
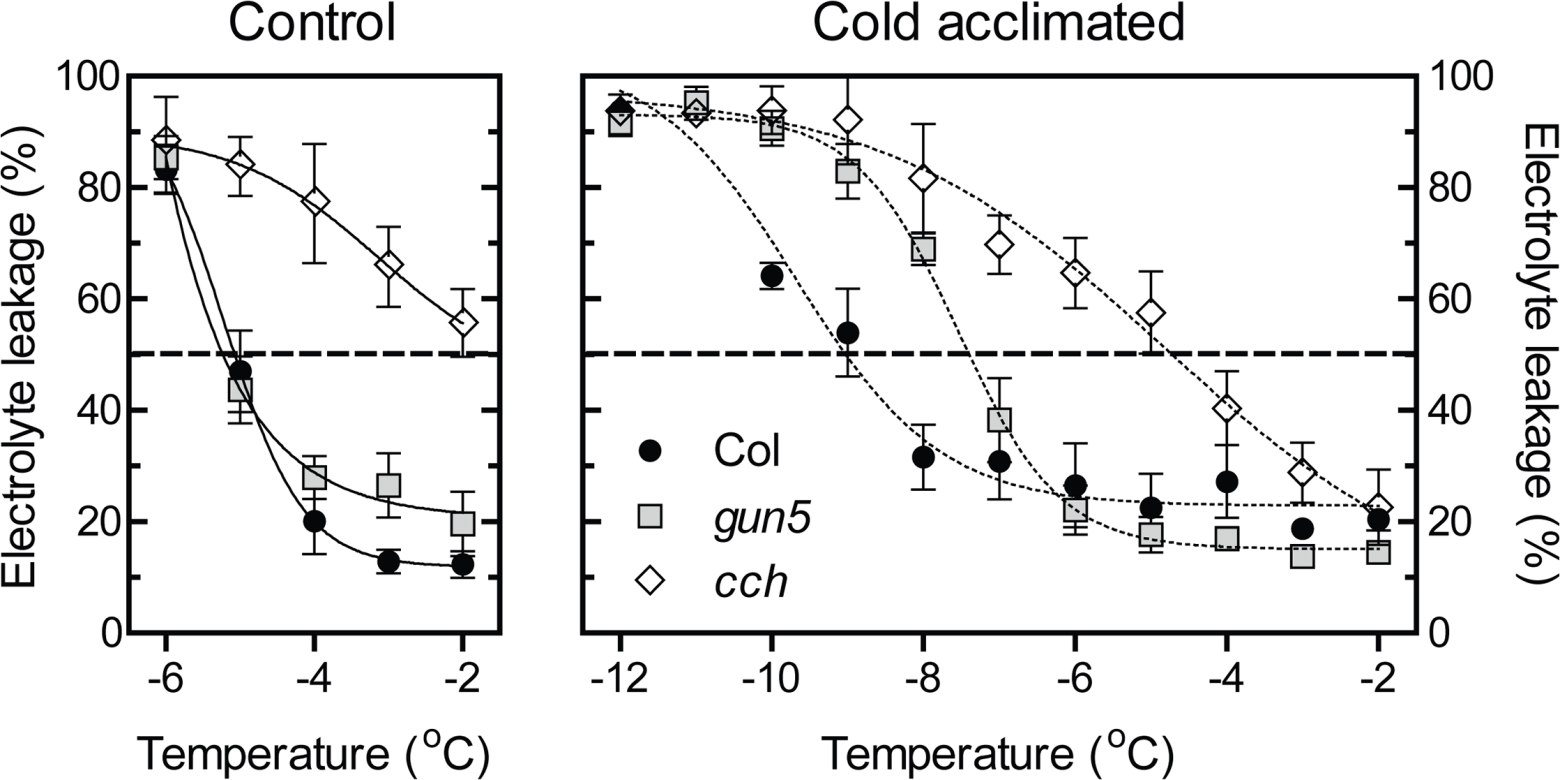
Freezing test of acclimated and non-acclimated Col-0, *gun5* and *cch* plants. Plants were grown for 5 weeks in SD conditions (left panel, non-acclimated) and transferred to SD conditions, 4°C for 3 days (right panel, cold-acclimated). Leaf discs of respective plant were exposed to decreasing temperatures (2°C/h) and checked for electrolyte leakage. Each data point represents values from at least three biological replicates (±SD).

**Fig 6:**
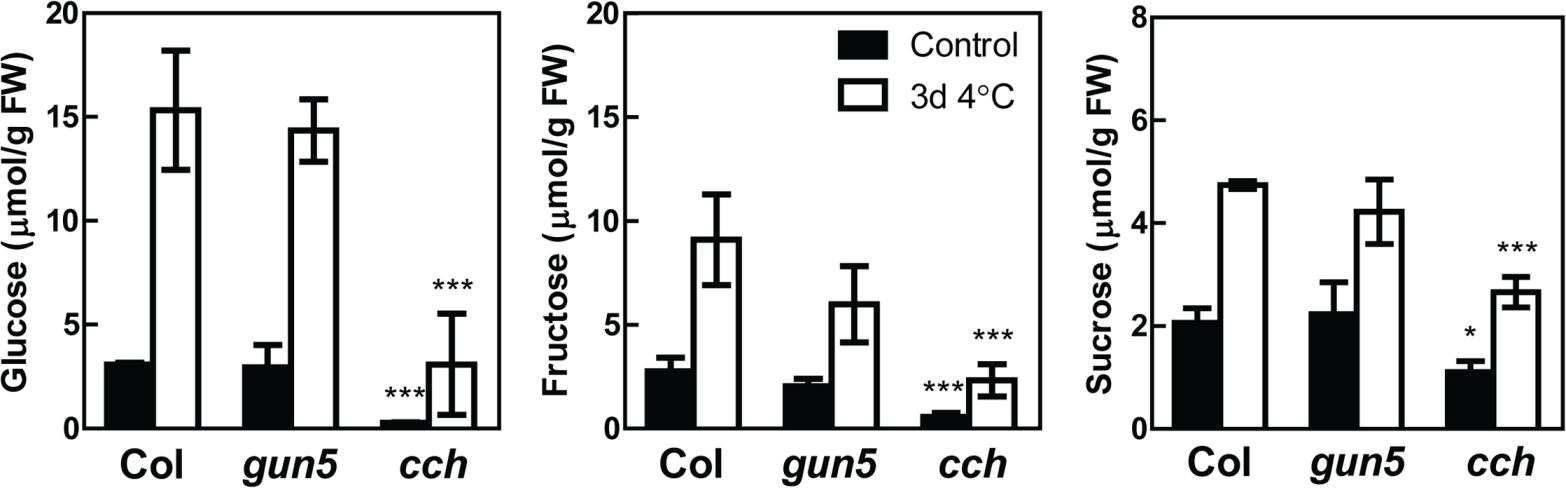
Sugar content in leafs of Col-0, *gun5* and *cch* grown under control conditions and following exposure to low temperature. 5 week old plants were sampled and checked for their leaf sugar content in control and after 3 days of cold exposure. Samples were taken in the middle of the light period. No significant differences could be seen between Col-0 and *gun5* plants (Student’s t-test). Between Col-0 and *cch*, significant differences could be seen for all sugars investigated, both in control conditions and following exposure to low temperature (Student’s t-test). p<0.05 (*), p<0.001 (***).

An electrolyte leakage test was performed on control plants and plants exposed to 4°C for 3 days in wild type, *gun5* and a stronger mutant allele of CHLH, *cch* (*c*onditional *ch*lorina [14]) (Fig 5). In *cch*, a C to T substitution results in a P642L mutation [14]. Similar to *gun5*, the mutant phenotype and the impaired plastid architecture of *cch* was enhanced when exposed to low temperatures (S1 Fig). No difference in freezing tolerance could be detected between wild type and *gun5* in non-acclimated control plants. The LT50 temperature was -4°C for both wild type and *gun5*. In contrast, the *cch* mutant showed a lower freezing tolerance in non-acclimated plants. After cold-acclimation for three days, wild type plants were considerably more tolerant to freezing temperatures compared to the *gun5* and *cch* plants. Wild type, *gun5* and *cch* plants showed 50% electrolyte leakage at temperatures of -9.7±0.2°C, -7.5±0.1°C and -4.8±0.7°C respectively. The experiment was repeated twice with similar results indicating that *gun5* plants are less freezing tolerant compared to wild type following cold acclimation. Although *cch* plants increased the freezing tolerance after cold acclimation, they have major difficulties to survive freezing temperatures, both in acclimated and non-acclimated plants compared to wild type. Soluble sugars are important as cryo-protectants [35]. To investigate any possible effect of lower amounts of sugars in the *gun5* and *cch* mutants, the levels of sucrose and free hexose were determined in plants grown under similar conditions as those used for the electrolyte leakage test (Fig 6). Acclimated and non-acclimated wild type and *gun5* plants showed no significant difference in their sugar content. Both genotypes displayed an increase of all sugars in response to low temperatures compared to control conditions. The *cch* mutant showed significantly lower amounts, in both control and cold acclimated plants, for all sugars measured (Fig 6). Thus *gun5* accumulates similar levels of soluble sugars compared to wild type following exposure to low temperatures indicating that the impaired freezing tolerance is not caused by lower levels of sugars. A more complex situation seems to be occurring in the *cch* mutant where the severity of the phenotype seems to have an impact on the ability to tolerate freezing temperatures.

### CHLH mutants have impaired protein translation in the cold

**Fig 7:**
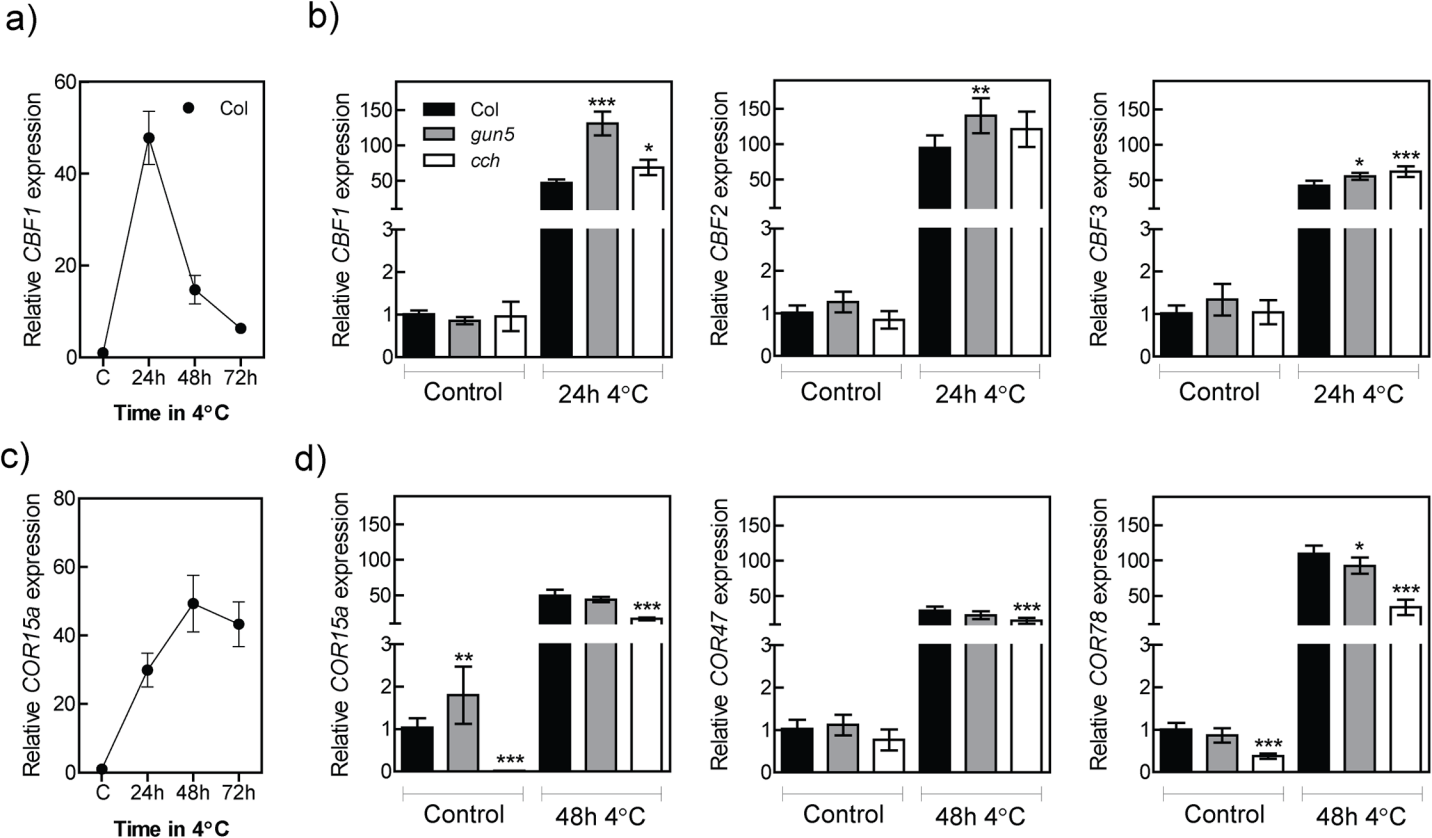
Transcript levels of *CBF1-3* and their downstream target, *COR15a* in Col-0, *gun5* and *cch* plants following cold exposure. 5-week old plants grown in SD conditions were transferred to SD conditions, 4°C, for indicated times. Extracted total RNA was DNase treated and cDNA synthesised. (a) Level of *CBF1* (At4g25490) transcript in Col-0 following exposure to 4°C. (b) Levels of *CBF1*, *CBF2* (At4g25470) and *CBF3* (At4g25480) transcripts in control conditions and after 24 hours of exposure to 4°C in Col-0, *gun5* and *cch* plants. (c) Level of *COR15a* (At2g42540) transcript in Col-0 following exposure to 4°C. (d) Levels of *COR15a*, *COR47* (At1g20440) and *COR78* (At5g52310) transcripts in control conditions and after 48 hours of exposure to 4°C in Col-0, *gun5* and *cch* plants. All transcript levels following exposure to 4°C were related to the transcript levels of respective grown control. Ubiquitin-like protein (At4g36800) was used as internal control. Data is from at least 3 independent replicates and show the mean (±SD). Significant differences was determined with two-way ANOVA with Bonferroni post-tests, p<0.05 (*), p<0.01 (**), p<0.001 (***).

**Fig 8:**
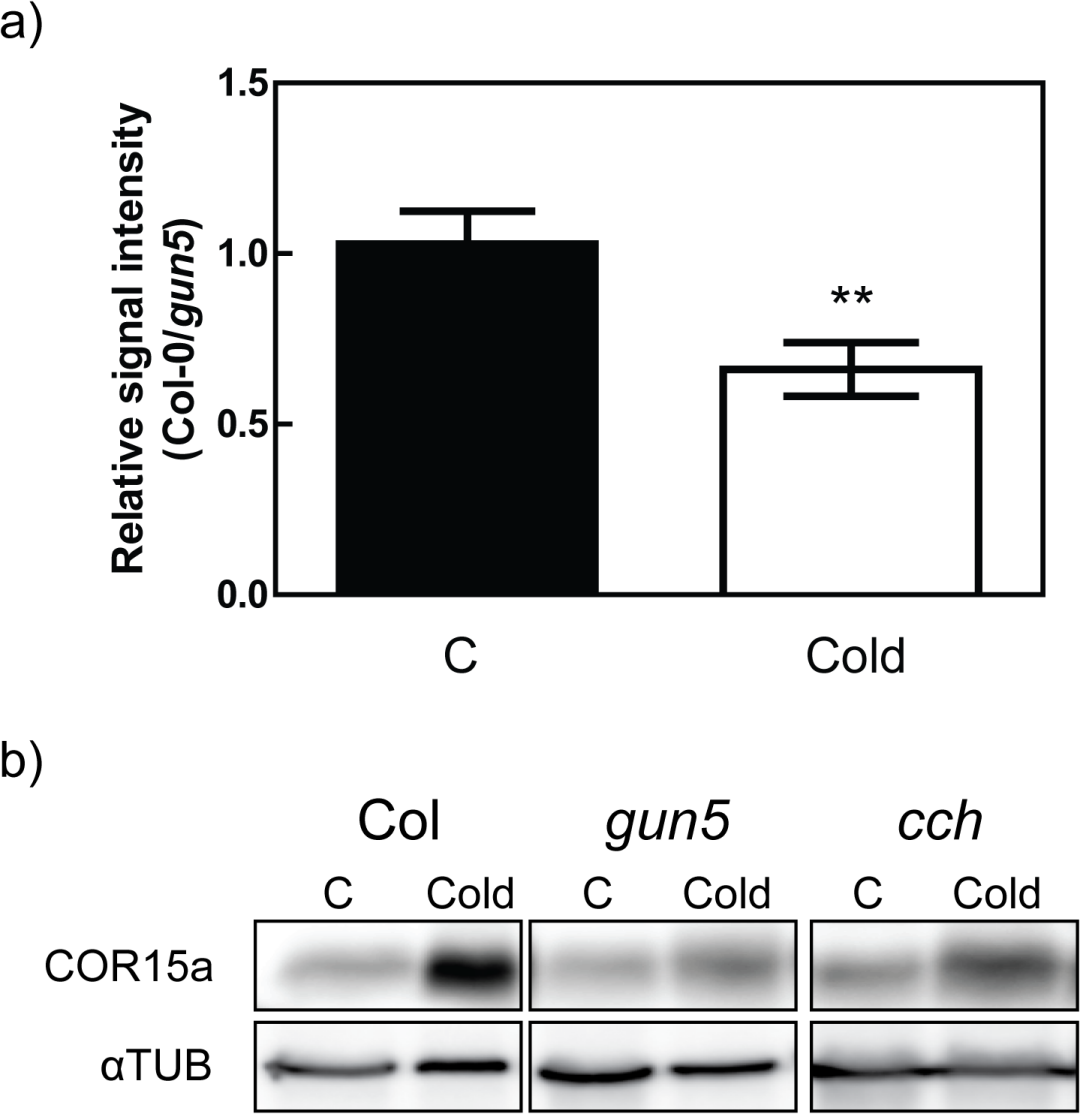
Protein synthesis is impaired in the CHLH mutants in response to low temperatures. (a) 3 week old plants were grown at SD conditions, 22°C, pre-treated at 0°C for 4 days and labelled with [35S]Met and [35S]Cys. The plants were then transferred to either 22°C or 0°C for 36 hours. Plants were briefly rinsed with water and total proteins were extracted and run on a SDS-PAGE. The gel was stained, dried and exposed to an X-ray film. Signal intensity of each lane was normalized to the intensity of the Coomassie stained gel and compared to the values of Col-0. Data is from 3 independent replicates and show the mean (±SD). (b) Western blot of COR15a after exposure to cold temperatures in Col *gun5* and the *cch* mutant. 5-week old plants were grown on soil in SD conditions and transferred to low temperature for 24 hours.

The C-repeat/dehydration-responsive element binding factors (CBFs) are important transcription factors for the cold acclimation process in *Arabidopsis* [4]. The *CBF* genes are upregulated within 15 min of exposure to low temperatures and high expression is maintained during the first 24 h in photoperiodic light (Fig 7A) [36]. To explore if altered expression levels of CBFs were responsible for the decreased freezing tolerance in the CHLH mutants *CBF1-3* expression was determined. Wild type showed an increase in *CBF1-3* expression after 24 hours followed by a decrease of the initial expression peak after 48 and 72 hours, a similar expression profile was observed in the *gun5* mutant (Fig 7A, S2 Fig). Intriguingly, both the *gun5* and *cch* mutants showed a stronger induction of *CBF1-3* expression compared to wild type after 24 h of low temperature exposure (Fig 7B). No significant difference between wild type, *gun5* and *cch* could be seen in control conditions (Fig 7B). To test if the super-induction of *CBF* expression in *gun5* and *cch* was relayed to their downstream targets, we checked the expression of *COR15a COR47* and *COR78*. COR15a is targeted to the chloroplast [37] while COR47 and COR78 are not [38]. The peak of expression for the *COR* genes occurs at 48 hours in our experimental conditions (Fig 7C, S2 Fig). The expression level of the CORs was surprisingly similar in wild type and *gun5* plants while the expression level in the *cch* mutant were generally lower compared to wild type, both in control and after 48 hours of low temperature exposure (Fig 7D). Thus, the super-induction of the CBFs is not reflected in the expression levels of the CORs. This could indicate that the translation or stability of the CBFs were somehow impaired in the CHLH mutants. To check the synthesis of new proteins in plants grown under control conditions and plants exposed to low temperature, wild type and *gun5* plants were incubated with 35S incorporated amino acids for 36 hours, proteins were extracted, run on a denaturing gel and exposed to an X-ray film. No difference in signal could be observed between wild type and *gun5* plants when grown under control conditions (Fig 8A, S3 Fig). In the cold treated plants however, *gun5* plants showed a decrease in the signal from newly synthesised proteins (Fig 8A, S3 Fig). To confirm the reduced synthesis of proteins in CHLH mutant plants exposed to low temperature, a COR15a antibody was used. Supporting our earlier results, both the *gun5* and the *cch* mutant showed a decreased level of COR15a protein when compared to wild type following exposure to low temperatures (Fig 8B). In summary, our results demonstrate that a functional chloroplast is essential for proper cold acclimation and suggest that an impaired chloroplast function could inhibit *de novo* protein synthesis at low temperature.

## Discussion

The CHLH mutants have clearly impaired ability to acclimate to low temperatures and as a consequence the mutants are more freezing sensitive compared to wild type. The inability to cold acclimate in the *gun5-1* (*gun5*) and the *cch* mutants is accompanied with a severely inhibited chlorophyll biosynthesis and reduced protein translation during low temperatures. Our results demonstrate the importance of a functional chloroplast and the recovery of photosynthesis for the cold acclimation process. The *gun5* mutation resides in a conserved residue of exon 3 in CHLH (A990V) [14]. Exposure to low temperature gave rise to an additional and more severe phenotype in *gun5* plants. Importantly, the enhanced phenotype of *gun5* plants was reversible and not caused by oxidative stress, a stress often accompanied with exposure to low temperatures, since cold treated *gun5* grown in very low light intensities showed a similar phenotype (Fig 1). Under our warm grown control conditions there was no difference in protein levels (Fig 3) or gene expression (Figs 3 and 7) between wild type and the *gun5* mutant. However, following exposure to low temperature significant changes were observed including a decreased freezing tolerance. Another allele of CHLH, *cch*, showed similar albeit more severe phenotype after exposure to low temperature (Fig 5, S1 Fig). Unlike the *gun5* mutant, *cch* showed major difficulties compared to wild type and *gun5* to survive freezing temperatures following growth under control conditions. The decline in freezing tolerance is likely a direct effect of the decreased synthesis of proteins observed in *gun5* (Fig 8). This is illustrated by the expression pattern of the CBFs, a super-induction that is not conveyed to their target genes (*COR15a*), and the overall decreased level of protein synthesis, in particular the level of cryo-protective peptides like COR15a (Fig 8). In the *cch* mutant, the situation seems to be more complex. The severity of the mutation results in affected plastid structure already in control conditions which, in turn, affect the levels of soluble sugars (Fig 6) and expression levels of *COR15a* and *COR78* (Fig 7). The *cch* mutant is also impaired in ABA signalling [24], discussed below, which almost certainly affect the ability to withstand freezing temperatures. It is interesting to note that the level of CHLH was not decreased in the *gun5* mutant following cold exposure (Fig 3). This suggests that the *gun5* mutation possibly affects the stability of the CHLH protein.

CHLH is localised in both soluble and membrane fractions of isolated plastids [39]. The association with the envelope and thylakoid membranes, where critical steps of chlorophyll synthesis occurs, is stimulated by bound substrate and GUN4 [22,39] as well as Mg^2+^ concentration [19]. Interestingly, the association with the membrane was shown to be inhibited by the mutated form of CHLH in the *gun5* and *cch* mutants [39]. Possibly this inhibition is enhanced by the low temperature which could explain the collapse of chlorophyll biosynthesis observed in *gun5* and *cch* at low temperatures (Fig 4). When inserted into the membrane, the N- and C-terminal parts of CHLH are in contact with the cytosol [19,40], placing the protein in a unique position to sense changes in chloroplastic membrane plasticity. Exposure to low temperatures initially decreases the membrane fluidity [41] and ought to disturb the association and disassociation of CHLH to the membrane. The main role of CHLH, biosynthesis of chlorophyll, is undoubtedly rapidly inhibited under low temperatures [34,42], (Fig 4). Following cold acclimation, there is an increase in phospholipids and the degree of unsaturation of fatty acids to enhance membrane fluidity [43]. This presumably leads to a recovery of chlorophyll biosynthesis seen in wild type after the initial inhibition (Fig 4). Consequently, the association of CHLH to the membrane, leading to an active chlorophyll biosynthesis, is pivotal for proper cold acclimation. Very little is known about how plastid defects affect cold acclimation but a functional chlorophyll biosynthesis is crucial for translation of PsbA [44] and the stability of LCH proteins [45]. Lower amounts of PsbA, PSAF and LHC proteins were observed in the *gun5* mutant compared to wild type following cold exposure and possibly the inability of the *gun5* mutant to recover synthesis of chlorophyll results in a decrease of plastid translation (Fig 3) which has been found to be important for cold acclimation [8,46–48]. Numerous reports demonstrate that impaired plastid transcription/translation effects nuclear gene expression [29,49,50]. This includes the impairment of the cold induced up-regulation of a sub-set of ribosomal genes in albino mutants of barley and wheat [51]. A possible direct link between the plastid status and cytosolic protein stability/translation has also been found. In the *vir*-*zb63* mutant of barley, the levels of the COR14b protein is determined by the plastid status without affecting the steady state level of the mRNA [52]. It is however unclear if it is a regulation of protein stability or translation efficiency. To our knowledge, no studies have previously shown that altered chlorophyll biosynthesis affects cytosolic protein translation.

Another possibility for the role of CHLH is as a temperature sensor or receptor in itself. CHLH has been reported to be an abscisic acid (ABA) receptor and mediates ABA signalling as a positive regulator [24]. ABA is a plant hormone with important roles in seed germination and seedling maturation as well as response to different environmental cues, including cold [53]. ABA levels increases in leaves during cold and many ABA responsive genes are also regulated by low temperatures [54]. Furthermore, ABA is capable of inducing *CBF* expression, albeit to a significantly lower degree compared to cold temperatures [55]. Recently, it was found that CHLH interacts with a set of WRKY transcription factors, an interaction that is ABA-dependent [19]. One of the identified proteins, WRKY40, interacts with the promoters of the CBFs and negatively regulates their expression [19]. The ABA receptor function of CHLH is however distinct from its role in chlorophyll biosynthesis and retrograde signalling [24]. Additionally, the *gun5-1* mutation was found to have wild type responses to ABA regarding seed germination and the mutated GUN5 protein did not bind ABA differently compared to wild type [24]. The *cch* mutant on the other hand, has a clear ABA-insensitive phenotype and the mutation result in less efficient binding of ABA [24]. It seems plausible that at least a part of the freezing sensitivity seen in the *cch* mutant is due to the inability to bind ABA. The ABA-receptor role of CHLH can therefore not fully be ruled out as an explanation for the freezing sensitivity of the CHLH mutants and additional experiments are required to find the mode-of-action of ABA in cold acclimation.

CHLH could potentially interact with other proteins than the described WRKY transcription factors. For the cold response, one candidate is LOS1, a translation elongation factor 2 [33]. LOS1 was found to directly interact with the product of CHLH, Mg-ProtoIX *in vitro* [56] and the molecular phenotype of a cold-sensitive allele, *los1-1*, resembles that of the *gun5* mutant [33]. Another candidate is suppressor of ABAR over-expressor (SOAR1), a RNA-binding protein that was genetically linked to CHLH and located in the cytosol and nucleus [57]. There is also a genetic link between CHLH and cytosolic heat shock protein 90 (HSP90) [18]. HSP90 has been found to accumulate in response to low temperature [58,59] and is important for protein maintenance and maturation [60,61]. However, further experiments are required to elucidate if CHLH acts directly as a sensor or indirectly via chlorophyll biosynthesis in the cold acclimation process.

The results presented here clearly show that CHLH activity and recovery of plastid function are essential for the cold response in *Arabidopsis*. This adds yet another role for CHLH, in addition to being an enzyme in chlorophyll biosynthesis, an ABA-receptor and an important component in plastid-to-nucleus signalling. Since the discovery of its importance in signalling 15 years ago [14], CHLH continues to be of particular interest for future research.

## Supporting Information

S1 Fig The *cch* mutant show enhanced phenotype after long term low temperature exposure. (PDF)

S2 Fig Expression of *CBF1-3* and *COR15a* in Col-0 and the *gun5* mutant. (PDF)

S3 Fig Gel images of the 35S experiment. (PDF)

S1 Table Primers used in this study. (PDF)
