# Supplementary material for: The Recovery of Plastid Function Is Required for Optimal Response to Low Temperatures in Arabidopsis
Source: PLoS One. 2015 Sep 14;10(9):e0138010. doi: 10.1371/journal.pone.0138010 (PMC4569060; doi:10.1371/journal.pone.0138010)
Supplement: S1 Fig — (PDF) [file pone.0138010.s001.pdf]

S1 Fig

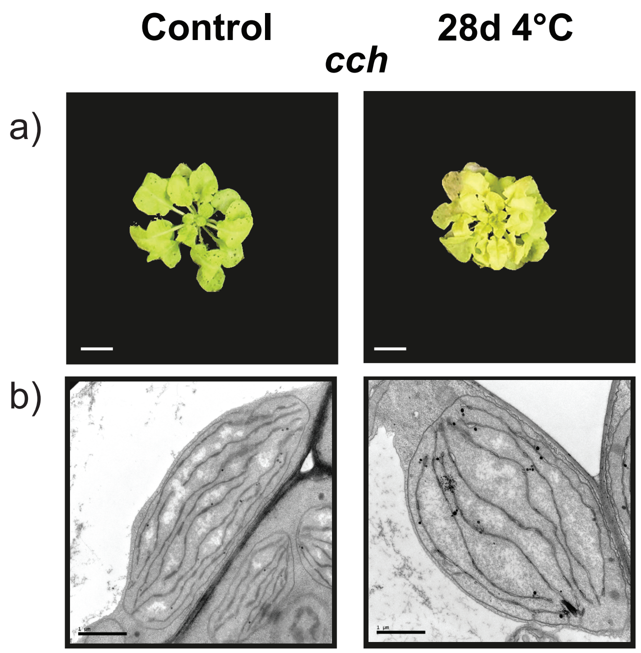

S1 Fig. The *cch* mutant show enhanced phenotype after long term low temperature exposure.
(a) 5 week old *cch* plants grown in short day condition (9/15 h light/dark, 22°C/18°C, 150 µE, control conditions) were transferred to 4°C, short day condition (9/15 h light/dark, 4°C/4°C, 150 µE) for 28 days. Bar represents 1 cm.
(b) Transmission electron microscopy (TEM) images of plastids from corresponding plants above. Bar represents 1 µm.
