# Supplementary material for: The Recovery of Plastid Function Is Required for Optimal Response to Low Temperatures in Arabidopsis
Source: PLoS One. 2015 Sep 14;10(9):e0138010. doi: 10.1371/journal.pone.0138010 (PMC4569060; doi:10.1371/journal.pone.0138010)
Supplement: S2 Fig — (PDF) [file pone.0138010.s002.pdf]

S2 Fig

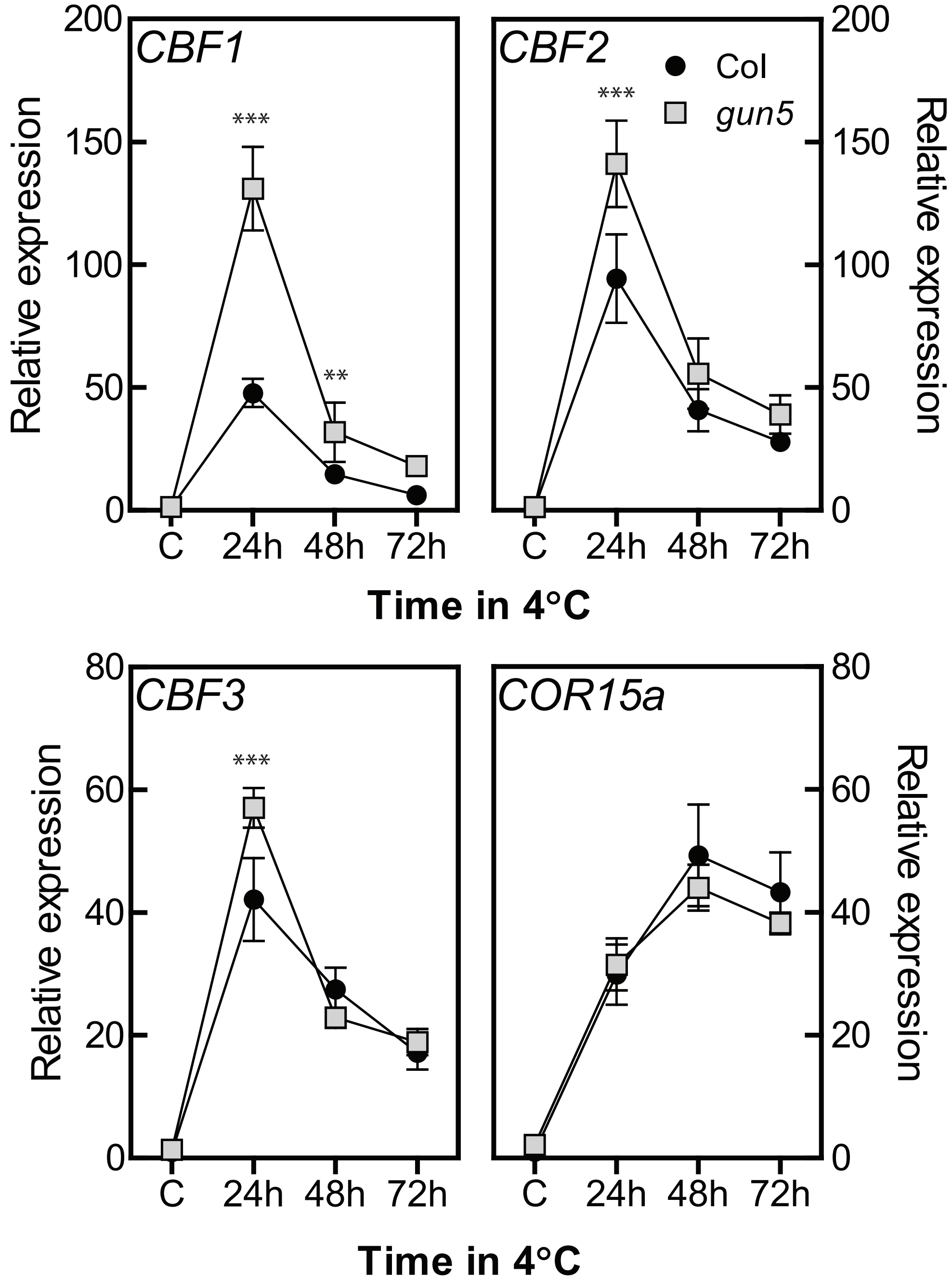

S2 Fig. Expression of *CBF1-3* and *COR15a* in Col-0 and the *gun5* mutant.
5-week old plants grown in SD conditions were transferred to SD conditions, 4°C, for indicated times. Extracted total RNA was DNase treated and cDNA synthesised. Levels of *CBF1* (At4g25490), *CBF2* (At4g25470), *CBF3* (At4g25480) and *COR15a* (At2g42540) were related to the transcript levels of the respective warm grown control. Ubiquitin-like protein (At4g36800) was used as internal control. Data is from at least 3 independent replicates and show mean (± SD). Significant differences were determined with two-way ANOVA with Bonferroni post-tests, p<0.01 (**), p<0.001 (***).
