# Supplementary material for: The Recovery of Plastid Function Is Required for Optimal Response to Low Temperatures in Arabidopsis
Source: PLoS One. 2015 Sep 14;10(9):e0138010. doi: 10.1371/journal.pone.0138010 (PMC4569060; doi:10.1371/journal.pone.0138010)
Supplement: S3 Fig — (PDF) [file pone.0138010.s003.pdf]

S3 Fig

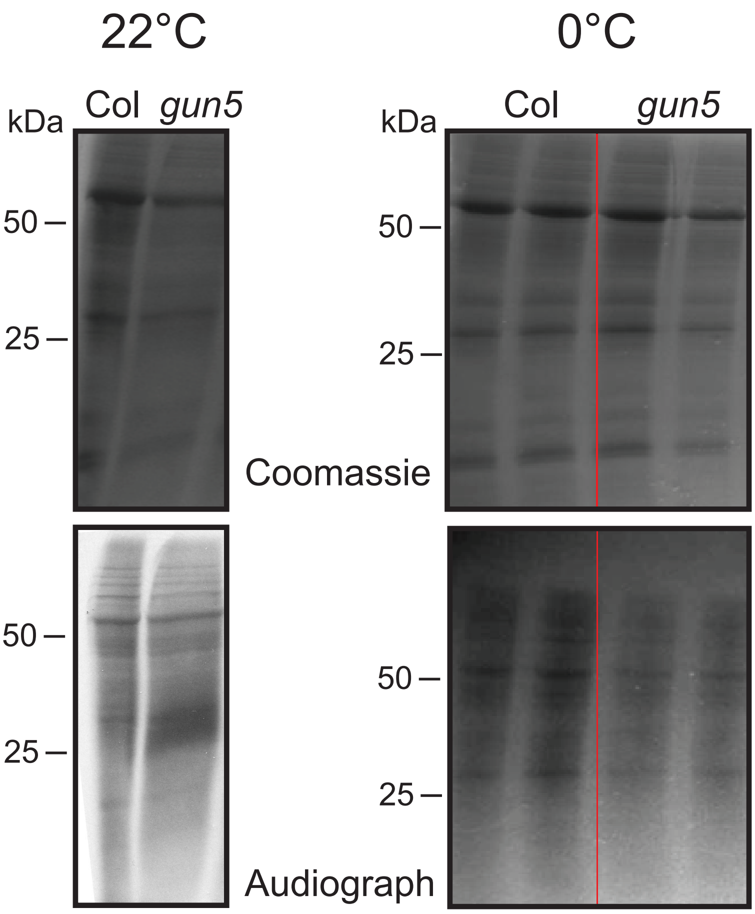

S3 Fig. Gel images of the 35S experiment.
3 week old plants were grown at SD conditions, 22°C, pretreated at 0°C for 4 days and labelled with [35S]Met and [35S]Cys. The plants were then transferred to either 22°C or 0°C for 36 hours. Plants were briefly rinsed with water and total proteins were extracted and run on a SDS-PAGE. The gel was stained (top panels), dried, exposed to an X-ray film and developed (bottom panels).
