# Supplementary material for: The Recovery of Plastid Function Is Required for Optimal Response to Low Temperatures in Arabidopsis
Source: PLoS One. 2015 Sep 14;10(9):e0138010. doi: 10.1371/journal.pone.0138010 (PMC4569060; doi:10.1371/journal.pone.0138010)
Supplement: S1 Table — (PDF) [file pone.0138010.s004.pdf]

Supplementary table 1. Primers used in this study

Gene	Locus	Forward primer	Reverse primer
CBF1	**AT4G25490**	GCCAAACAAGAAAACCAGGA	TCAGCGAAGTTGAGACATGC
CBF2	**AT4G25470**	TGACGTGTCCTTATGGAGCTA	CTGCACTCAAAAACATTTGCA
CBF3	**AT4G25480**	GATGACGACGTATCGTTATGGA	TACACTCGTTTCTCAGTTTTACAAAC
COR15a	**AT2G42540**	AACGAGGCCACAAAGAAAGC	CAGCTTCTTTACCCAATGTATCTGC
COR47	**AT1G20440**	ACAAGCCTACTGTCATCGAAAAGC	TCTTCATCGCTCGAAGAGGAAG
COR78	**AT5G52310**	GATGCACCAGGCGTAACAGGT	GTCCTTACAGAATGAGCCGG
LHCB1.1	**AT1G29920**	CGGAAAGTGAGCCAAGTTCT	TGAAAGTCTCTACCATCCACCA
LHCB2.4	**AT3G27690**	GCCATCCAACGATCTCCTC	TGGTCCGTACCAGATGCTC
GUN4	**AT3G59400**	CTTACACACACCACCGCAAC	AGGTGGAAGAAGCAGAGCAG
CHLH	**AT5G13630**	CCTAGAACAGGGAAGTCACA	CAGGTGGGAAACTGAAAACAG
PsbA	**ATCG00020**	ATACAACGGCGGTCCTTATG	GTATGCGTCCTTGGATTGCT
